# *Listeria monocytogenes* Associated with Pasteurized Chocolate Milk, Ontario, Canada

**DOI:** 10.3201/eid2503.180742

**Published:** 2019-03

**Authors:** Heather Hanson, Yvonne Whitfield, Christina Lee, Tina Badiani, Carolyn Minielly, Jillian Fenik, Tony Makrostergios, Christine Kopko, Anna Majury, Elizabeth Hillyer, Lisa Fortuna, Anne Maki, Allana Murphy, Marina Lombos, Sandra Zittermann, Yang Yu, Kristin Hill, Adrienne Kong, Davendra Sharma, Bryna Warshawsky

**Affiliations:** Public Health Ontario, Toronto, Ontario, Canada (H. Hanson, Y. Whitfield, C. Lee, T. Badiani, A. Majury, L. Fortuna, A. Maki, A. Murphy, M. Lombos, S. Zittermann, Y. Yu, B. Warshawsky);; Simcoe Muskoka District Health Unit, Barrie, Ontario, Canada (C. Minielly, J. Fenik, T. Makrostergios);; Canadian Food Inspection Agency, Ottawa, Ontario, Canada (C. Kopko, K. Hill, A. Kong, D. Sharma);; Public Health Agency of Canada, Guelph, Ontario, Canada (E. Hillyer);; Western University, London, Ontario, Canada (B. Warshawsky)

**Keywords:** Listeria monocytogenes, bacteria, listeriosis, outbreak, chocolate milk, pasteurization, enteric infections, food safety, whole-genome sequencing, pulsed-field gel electrophoresis, Ontario, Canada

## Abstract

In an investigation of a listeriosis outbreak in Ontario, Canada, during November 2015–June 2016, pasteurized chocolate milk was identified as the source. Because listeriosis outbreaks associated with pasteurized milk are rare in North America, these findings highlight that dairy products can be contaminated after pasteurization.

*Listeria monocytogenes* is a formidable pathogen acquired primarily through contaminated food. Invasive listeriosis is a reportable disease in Ontario, Canada; ≈50 case-patients (0.4 cases/100,000 persons) have been reported annually since 2005 (*1*). Recent outbreaks of listeriosis in North America have been associated with delicatessen meats, soft cheeses, raw produce, and unpasteurized dairy products (*2**–**4*). However, listeriosis outbreaks linked to pasteurized fluid milk are rare.

A study in the United States reviewed 83 fluid milk–associated disease outbreaks during 1990–2006; however, only 1 outbreak was attributed to *L. monocytogenes* (*5*). We report an outbreak of listeriosis associated with pasteurized chocolate milk in Ontario, Canada.

## The Study

**Figure 1:**
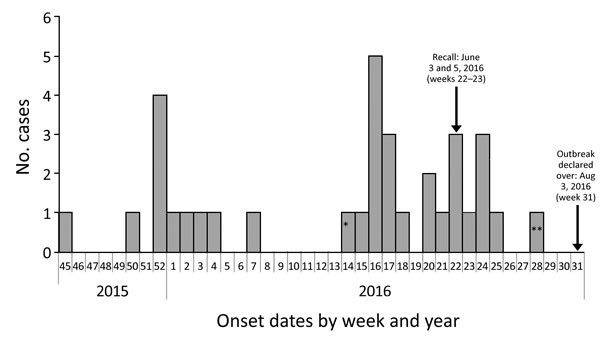
Outbreak cases of listeriosis (n = 34) by onset week and year, Ontario, Canada, November 2015–August 2016. Data were obtained from the Ontario Ministry of Health and Long-Term Care, integrated Public Health Information System database, extracted by Public Health Ontario, August 16, 2016. Weeks are defined according to the Public Health Agency of Canada epidemiologic week calendar. *Neonatal case-patient with symptom onset on April 4, 2016 (week 14), and illness most likely caused by mother-to-child transmission. **Asymptomatic case-patient from whom a specimen was collected on July 13, 2016, and exposure occurred before June 27, 2016 (week 28).

We defined an outbreak case-patient as a person in Ontario with listeriosis symptom onset after November 1, 2015, who had pulsed-field gel electrophoresis (PFGE) pattern combinations LMACI.0015/LMAAI.0024 or LMACI.0015/LMAAI.0069. Thirty-four case-patients met the outbreak definition; only Ontario residents were identified. Eleven case-patients had an onset date during November 14, 2015–February 14, 2016. Onset dates ranged from April 11 to June 20, 2016, for 21 case-patients in the second wave; the remaining 2 case-patients were outliers (Figure 1). Median age was 73 years (range <1–90 years). More than half of the case-patients were female (20/34, 59%). Hospitalizations occurred for 32 (94%) case-patients, and 4 deaths (12%) were reported.

In Ontario, local public health professionals complete the national invasive listeriosis questionnaire and collect food samples. We conducted a case–case analysis by using Ontario case-patients listed in the national listeriosis database as controls. We used a variety of methods to support hypothesis generation, including supplemental questionnaires, centralized interviewing, and reviewing purchase records collected through shoppers’ loyalty card programs. A meeting was also held with representatives from a grocery chain that was common for case-patients (retail chain A) for insights into possible sources.

PFGE and whole-genome sequencing were performed at the Public Health Ontario Laboratory, in accordance with PulseNet Canada protocols (Table). Food safety investigations, including targeted retail sampling, were conducted by the Canadian Food Inspection Agency and Ontario Ministry of Agriculture, Food and Rural Affairs. Laboratory analyses of food samples were conducted by the Canadian Food Inspection Agency and the Public Health Ontario Laboratory.

**Table:** Characteristics of 23 *Listeria monocytogenes* isolates analyzed by whole-genome sequencing during listeriosis outbreak in pasteurized chocolate milk, Ontario, Canada*

Isolate ID	Isolate source	PFGE pattern, first enzyme/second enzyme	SRA accession no.
ON-1501	Human	LMACI.0015/LMAAI.0069	SAMN09909078
ON-1502	Human	LMACI.0015/LMAAI.0024	SAMN09909079
ON-1503	Human	LMACI.0015/LMAAI.0024	SAMN09909080
ON-1601	Human	LMACI.0015/LMAAI.0069	SAMN09909081
ON-1602	Human	LMACI.0015/LMAAI.0024	SAMN09909082
ON-1603	Human	LMACI.0015/LMAAI.0069	SAMN09909083
ON-1604	Human	LMACI.0015/LMAAI.0024	SAMN09909084
ON-1605	Human	LMACI.0015/LMAAI.0069	SAMN09909085
ON-1606	Human	LMACI.0015/LMAAI.0069	SAMN09909086
ON-1607	Human	LMACI.0015/LMAAI.0069	SAMN09909087
ON-1608	Human	LMACI.0015/LMAAI.0024	SAMN09909088
ON-1609	Human	LMACI.0015/LMAAI.0069	SAMN09909089
ON-1610	Human	LMACI.0015/LMAAI.0024	SAMN09909090
ON-1611	Human	LMACI.0015/LMAAI.0069	SAMN09909091
ON-1612	Human	LMACI.0015/LMAAI.0024	SAMN09909092
ON-1613	Human	LMACI.0015/LMAAI.0069	SAMN09909093
ON-1614	Human	LMACI.0015/LMAAI.0069	SAMN09909094
ON-1615	Human	LMACI.0015/LMAAI.0069	SAMN09909095
ON-1616	Human	LMACI.0015/LMAAI.0069	SAMN09909096
ON-1617	Human	LMACI.0015/LMAAI.0069	SAMN09909097
ON-1618	Human	LMACI.0015/LMAAI.0069	SAMN09909098
ON-1619	Human	LMACI.0015/LMAAI.0069	SAMN09909099
ON-1620	Human	LMACI.0015/LMAAI.0069	SAMN09909100
ON-1621	Human	LMACI.0015/LMAAI.0069	SAMN09909101
ON-1622	Human	LMACI.0015/LMAAI.0069	SAMN09909102
ON-1623	Chocolate milk	LMACI.0015/LMAAI.0069	SAMN09909103

*All strains were from Bioproject PRJNA486837. ID, identification; ON, Ontario; PFGE, pulsed-field gel electrophoresis; SRA, sequence read archive.

Several hypotheses were generated during the course of this outbreak. In the first wave, a concurrent listeriosis outbreak associated with leafy greens was ongoing in the United States and Canada. However, product testing did not establish a relationship between the 2 outbreaks. Cheddar cheese was also suspected, but a food safety investigation, including sampling at the manufacturer, did not support a link to this outbreak (*6**,**7*). Although leafy greens and cheddar cheese were ruled out, 1 commonality remained; shopping at retail chain A was reported frequently by case-patients.

A second wave began in April 2016 in which 10 of 17 case-patients reported consuming coleslaw. Six case-patients ate coleslaw from the same manufacturer, which supplied retail chain A and a fast food restaurant chain. However, the food safety investigation, including sampling at the manufacuturer and supplier, did not support this hypothesis.

**Figure 2:**
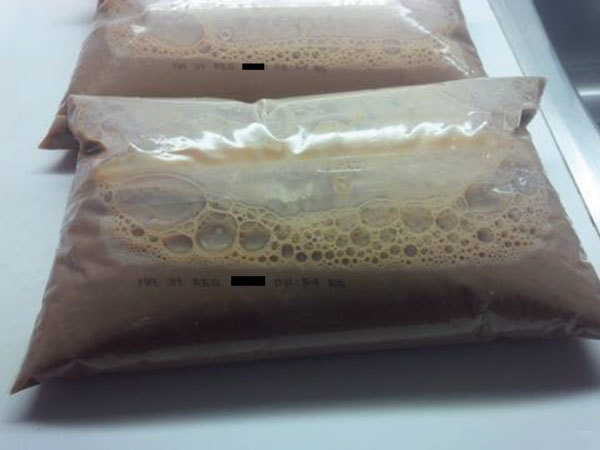
Bags of pasteurized chocolate milk as sold in Canada, with outer bag containing brand information removed. A bag of milk similar to these, found at the home of 1 case-patient during investigation of an outbreak of *Listeria monocytogenes* infection associated with pasteurized chocolate milk in Ontario, Canada, was found to be contaminated with the same strain obtained from infected patients.

On May 24, 2016, *L. monocytogenes* isolated from expired bagged chocolate milk collected from the home of 1 case-patient was confirmed to have the outbreak strain PFGE pattern. Fluid milk in Canada is often sold in plastic bags (Figure 2). In this instance, the outer packaging, which is the only area that contains the brand name, was discarded. Thus, the brand name was uncertain, and efforts were undertaken to confirm the source of the chocolate milk. Because the proxy of the case-patient reported purchasing brand B milk, samples of brand B chocolate and white milk were collected from retail for testing. Brand B was the main brand of chocolate milk sold by retail chain A, and it is distributed primarily in Ontario.

Although the hypothesis-generating questionnaire used stipulated milk, with flavored milk as a prompt, chocolate milk was not specified, and as a result this type of milk might have been underreported. Exposure to pasteurized milk was reported by 60% of case-patients in the first wave compared with 76% of controls. Thus, milk was not originally pursued as a source. However, this new positive isolate led to reinterviewing of case-patients from the second wave and resulted in 9 (75%) of 12 case-patients reporting consuming brand B when asked specifically about chocolate milk.

On June 3, a retail sample of brand B chocolate milk produced at facility C was confirmed positive for *L. monocytogenes*. This finding led to a class I recall of 1 lot of brand B chocolate milk. On June 5, the recall was expanded to all lots of brand B chocolate milk processed at that facility which only distributes in Ontario. Isolates from the original sample and 3 subsequent positive samples of chocolate milk, obtained from extensive retail sampling, matched the outbreak strain by PFGE and whole-genome sequencing. No white milk samples were positive for *L. monocytogenes*.

Environmental sampling at the manufacturer confirmed the presence of the outbreak strain within a postpasteurization pump dedicated to chocolate milk and on nonfood contact surfaces. This postprocess contamination of the chocolate milk line was believed to be the root cause of the outbreak. A harborage site might have been introduced by a specific maintenance event or poor equipment design. The equipment was subsequently replaced, and corrective measures were implemented to prevent recurrence. Chocolate milk production was resumed after rigorous testing for *L. monocytogenes* under regulatory oversight.

## Conclusions

This outbreak lasted 7 months and resulted in 34 confirmed listeriosis case-patients. Discovering the cause of this listeriosis outbreak was challenging because pasteurized chocolate milk is a commonly consumed product. Although there have been previous outbreaks outside Canada caused by chocolate milk (*8*), pasteurized milk products are generally not expected to be the source. This outbreak highlights that even pasteurized products can be contaminated by and support the proliferation of *L. monocytogenes* when contamination is introduced postpasteurization. The possibility of postprocessing contamination indicates an ongoing need for regulatory oversight and robust quality assurance processes, which include routine sampling of the environment and finished products.

Brand B chocolate milk is a widely distributed product in Ontario, and contamination of this product could have resulted in >34 case-patients. It is possible that a lower number of case-patients were reported because chocolate milk may primarily be consumed by younger, healthier persons, in whom invasive listeriosis is less likely to develop (*9*). Another possible explanation is that the contamination in the milk appeared to be intermittent, with some samples testing positive and others testing negative. As such, careful attention should be given to equipment design and maintenance programs, as harborage sites could result in recurring contamination that goes undetected by routine monitoring. Targeted retail and environmental sampling was instrumental in identifying the root cause in the facility and the breadth of potentially implicated products in the marketplace. Thus, this type of sampling should be considered during outbreak investigations.

Ultimately, the implicated product was determined on the basis of testing of food items obtained from the home of 1 case-patient. This finding highlights the necessity of obtaining a thorough food history and collecting and testing available samples of food that case-patients consumed during the incubation period (*10*). In Canada, where bagged milk is common, labeling of the inner and outer bags with the brand name would facilitate product identification by consumers. This recommendation could extend to other food products in North America (e.g., frozen hamburger patties) that have multiple layers of packaging (*11*).

Appendix Additional information on *Listeria monocytogenes* associated with pasteurized chocolate milk, Ontario, Canada.
