# Supplementary material for: Listeria monocytogenes Associated with Pasteurized Chocolate Milk, Ontario, Canada
Source: Emerg Infect Dis. 2019 Mar;25(3):581–4. doi: 10.3201/eid2503.180742 (PMC6390750; doi:10.3201/eid2503.180742)
Supplement: Appendix — Additional information on Listeria monocytogenes associated with pasteurized chocolate milk, Ontario, Canada. [file 18-0742-Techapp-s1.pdf]

Article DOI: https://doi.org/10.3201/eid2503.180742

Listeria monocytogenes Associated with Pasteurized Chocolate Milk, Ontario, Canada

Appendix

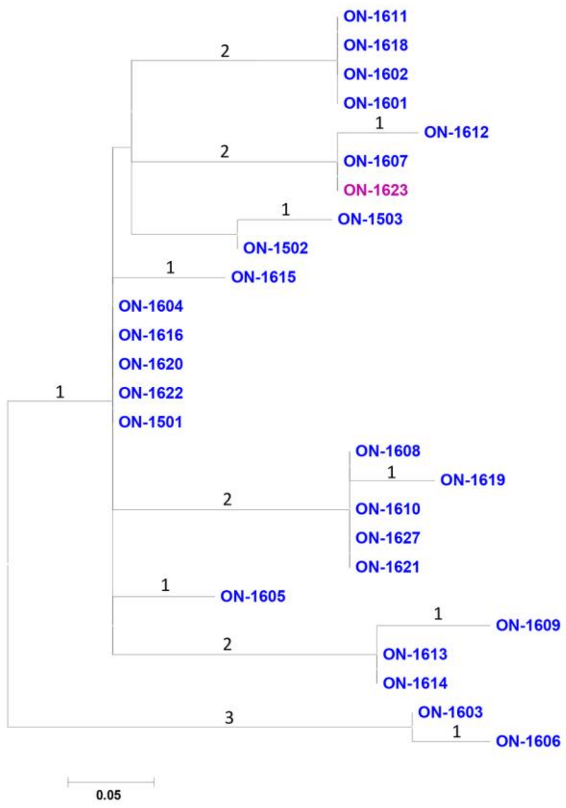

Appendix Figure. Maximum-likelihood tree for 26 *Listeria monocytogenes* isolates analyzed during listeriosis outbreak, Ontario, Canada. Isolates from clinical specimens fitting the outbreak definition (blue) and an isolate from chocolate milk from a patient's home (purple) were sequenced by using an Illumina (https://www.illumina.com/) MiSeq instrument and mapped to the de novo assembly of isolate ON-1603. A custom pipeline was used to identify high-quality core single-nucleotide variants (SNVs). Comparison yielded 20 SNVs. Numbers along branches indicate number of SNVs between nodes. Scale bar indicates nucleotide substitutions per site. ON, Ontario.
